# Comparative Genomics and Disorder Prediction Identify Biologically Relevant SH3 Protein Interactions

**DOI:** 10.1371/journal.pcbi.0010026

**Published:** 2005-08-12

**Authors:** Pedro Beltrao, Luis Serrano

**Affiliations:** EMBL Structural and Computational Biology, Heidelberg, Germany; Yale University, United States of America

## Abstract

Protein interaction networks are an important part of the post-genomic effort to integrate a part-list view of the cell into system-level understanding. Using a set of 11 yeast genomes we show that combining comparative genomics and secondary structure information greatly increases consensus-based prediction of SH3 targets. Benchmarking of our method against positive and negative standards gave 83% accuracy with 26% coverage. The concept of an optimal divergence time for effective comparative genomics studies was analyzed, demonstrating that genomes of species that diverged very recently from *Saccharomyces cerevisiae* *(S. mikatae, S. bayanus,* and *S. paradoxus),* or a long time ago *(Neurospora crassa* and *Schizosaccharomyces pombe),* contain less information for accurate prediction of SH3 targets than species within the optimal divergence time proposed. We also show here that intrinsically disordered SH3 domain targets are more probable sites of interaction than equivalent sites within ordered regions. Our findings highlight several novel *S. cerevisiae* SH3 protein interactions, the value of selection of optimal divergence times in comparative genomics studies, and the importance of intrinsic disorder for protein interactions. Based on our results we propose novel roles for the *S. cerevisiae* proteins Abp1p in endocytosis and Hse1p in endosome protein sorting.

## Synopsis

How can we tackle the complexity of a living cell? It is commonly said that living organisms are complex and display “emergent” properties. Emergence is perceived in this context as behaviors that appear at the system level but are not observable at the level of the system's components. In the cell this would be equivalent to saying that the cellular complexity could be explained if we could understand the interplay between the cellular components: that is, not just describe the “parts” that make up a cell but understand how they interact with each other to perform the necessary tasks.

A big step on the road to understanding cellular complexity will be a complete list of all relevant interactions between the cellular components. Although a lot of progress as been made in this direction, we are often dependent on experimental methods that are costly and time consuming. It's a big challenge for computational biology to process the current available knowledge and to propose new ways of predicting the interactions between cellular components.

Here the researchers studied protein interactions that are mediated by small linear peptide motifs,specifically interactions between a protein's SH3 domain and its targets, usually small peptide stretches containing a PXXP motif (where P is proline and X is any amino acid). The results showed that the putative target motifs that are conserved in ortholog proteins and are within regions that do not have a defined secondary structure are more likely to be relevant binding sites. Besides proposing a way to combine secondary structure information with comparative genomics to predict protein–protein interactions, the researchers highlight a possible role of intrinsically disordered proteins in SH3 protein interactions. The results also show that when looking for conservation of these motifs, it is important to carefully select the species used in the study: comparisons between species that have diverged to a certain extent—not too little and not too much—are the most informative.

## Introduction

Important advances have been made in using computational methods to mine the ever-growing quantity of experimental results in order to derive predictions of protein–protein interactions. For such interactions there are methods that explore sequence and structure analysis, like gene fusion [1,2], gene order [3], phylogenetic profiling [4–7], correlated mutations [8,9] and multimeric threading [10,11]. It as also been shown that it is possible to combine different experimental and functional data to predict protein interactions, especially when weighted using Bayesian networks [12]. The accumulation of validated interactions can also be mined by interlog mapping in order to transfer protein interaction annotations across species [13,14].

The work described here deals with the prediction of protein interactions mediated by recognition modules that target small linear motifs [15,16] and more specifically interactions involving SH3 domains. This type of asymmetric binding between globular domains and linear peptides was first reported in the work on Src kinase [17–20], and many other domains have now been shown to have similar properties [15,16]. In a previous study [21], knowledge from phage display experiments was used to derive a position-specific scoring matrix (PSSM) for particular SH3 domains, which was then used to predict putative target ligands. Later, Tong et al. devised a strategy where two-hybrid screening and PSSM were combined to derive a high-confidence network [22]. It was reasoned that an interaction identified by two-hybrid screening was more likely to be biologically relevant if the target protein had a high-scoring linear peptide according to the PSSM of the bait SH3 domain.

In this work we set out to obtain a high-confidence, biologically relevant protein interaction network, starting from the consensus information and using computational methods. The study showed that it is possible to greatly increase the accuracy of consensus-based predictions of protein–linear sequence interactions by taking into consideration the fact that biologically relevant target ligands of SH3 domains are more likely to be within disordered regions and conserved in orthologs. The method's performance was improved by selection of species within an optimal divergence time from the species of interest.

It has been proposed that intrinsic disorder may play a role in protein interactions [23–26], and there are documented cases where binding is coupled to folding [27,28] (reviewed in [29]). It has also been observed that small linear motifs tend to accumulate in protein regions predicted to be intrinsically disordered [30] and that proline-rich regions are usually devoid of secondary structure [31]. In most structures that we are aware of, the SH3 domain is in complex only with short target peptides, and not with full proteins. In all cases the ligands adopt a nonregular secondary structure, but there is little information one can take from these, in respect to the order/disorder of target sites in the context of the whole target protein. Although there is currently no experimental evidence to support that the SH3 domains preferentially bind to intrinsically disordered regions, the results presented here show that binding motifs within disordered protein regions are more likely to be biologically relevant binding sites than equivalent sites within ordered regions. We use the method developed to suggest novel SH3 interactions for *Saccharomyces cerevisiae* and provide information about the binding sites within the target proteins.

## Results/Discussion

### Identification and Conservation of SH3 Domains and Selection of Genomes

**Figure 1:**
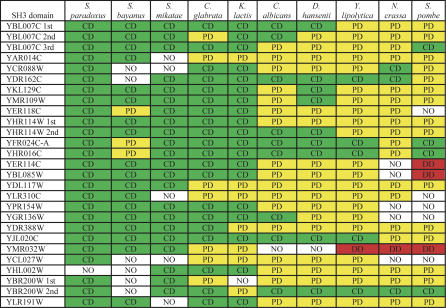
Conservation Study of the SH3 Domains of *S. cerevisiae* in Ten Other Yeast Genomes CD, conserved domain (the SH3-containing protein has an ortholog and the ortholog SH3 domain is possibly conserved, i.e., less than three conservative changes and no nonconservative changes in the binding positions); DD, divergent domain (SH3-containing protein has an ortholog in this genome but the domain is not on the same branch of the phylogenetic tree); NO, no ortholog (no ortholog found for SH3-containing protein in a particular genome); PD, possibly divergent (SH3-containing protein has an ortholog in this genome but the ortholog SH3 domain has at least one nonconservative change in the binding positions or more than two conservative changes in the binding positions).

Using profile hidden Markov models (see Materials and Methods; Figure 1), all putative SH3 domains, and their key binding positions (see Materials and Methods) were determined in *S. cerevisiae* and in a set of thirteen yeast species: *Candida glabrata, Debaryomyces hansenii, Kluyveromyces lactis, Yarrowia lipolytica* [32], *C. albicans* [33], *S. paradoxus, S. bayanus, S. mikatae* [34], *S. castellii, S. kudriavzevii,* *S. kluyveri* [35], *Neurospora crassa* [36], and *Schizosaccharomyces pombe* [37].

In *S. castellii,* *S. kluyveri,* and *S. kudriavzevii,* no orthologs for the majority of the *S. cerevisiae* SH3 domains could be identified (results not shown). However, these genomes had only been sequenced with a 2- to 3-fold coverage [35], which may have led to some genomic regions being poorly sequenced. As a result of this, these three genomes were not included in our work.

The ortholog SH3 domains were split into three groups: conserved domain, possibly divergent (if the putative ortholog SH3 domain was in the same branch of the phylogenetic tree and had more than two conservative changes in the binding positions; see Materials and Methods), or divergent domain (if the putative ortholog SH3 domain was not in the same branch of the phylogenetic tree) (Figure 1). As expected, the percentage of conserved domains was higher in genomes of species that had diverged recently from *S. cerevisiae*.

Intuitively we can expect that there will be an optimal divergence time for the species used in a particular comparative genomic study. In recently diverged species, most protein sequence is conserved and the statistical power for comparative genomics of biological features is therefore smaller. Interspecies conservation becomes less meaningful in a background of low evolutionary divergence. On the other hand, finding a conserved consensus in a very divergent genome might be more significant but only if there was no major change in the specificity of the domain. This change will be more probable the more divergent the species is from the species of interest.

To test the improvement of consensus-based predictions with a comparative genomics approach, an initial set of genomes was chosen based on the conservation analysis of the SH3 domains across the different yeast species (Figure 1). *N. crassa* and *Sch. pombe* were excluded because the SH3 domains in these two species might be too divergent to observe conservation of the *S. cerevisiae* motifs. Very close relatives *(S. paradoxus, S. bayanus,* and *S. mikatae)* were excluded as these species would have lower statistical power. Therefore, the first group analyzed consisted of five yeast genomes that broadly covered the hemiascomycete phylum, containing the four recently reported genomes of *C. glabrata, D. hansenii, K. lactis,* and *Y. lipolytica* that we grouped with the *C. albicans* genome.

### Evaluation of the “Conservation” Approach

**Figure 2:**
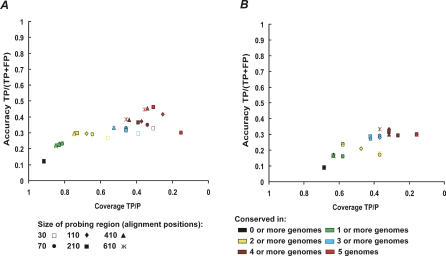
Size of Probing Window When Looking for Conservation of the Consensus Sequence in Orthologs of the Putative Target Protein We defined the conservation score as simply the number of species where the consensus sequence is conserved. With this information the accuracy and coverage were calculated, with the gold (A) and platinum (B) positive sets, for consensus sequence conserved in different numbers of species and for different sizes of the probing region.

To evaluate the predictive power of our method, two positive datasets, containing experimentally verified SH3–linear peptide interactions, and one negative dataset, containing noninteracting protein pairs were defined (see Materials and Methods). The binding motifs of the SH3 domains of *S. cerevisiae* included in the two sets of positive standards (15 SH3 domains in the gold set and ten in the platinum set) were taken from the data published by Tong et al. [22]. Table 1 shows the consensus sequence used in the study and also, for each SH3 domain, the total number of peptides found matching this sequence in the *S. cerevisiae* proteome. From this a measure of accuracy and coverage (see Materials and Methods) based on the positive and negative datasets was calculated. For simple pattern matching of consensus sequence, the accuracy (defined as TP/[TP + FP], where TP indicates true positives and FP indicates false positives) for predicting protein interaction was 12% and the coverage (defined as TP/P, where P indicates all positives) was 92% when using the gold positives set (see Figure 2A).

**Table 1:**
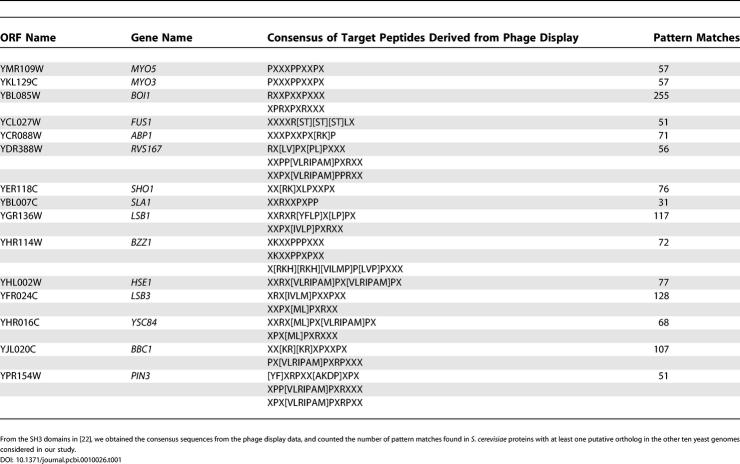
SH3 Consensus Sequence Information

From the SH3 domains in [22], we obtained the consensus sequences from the phage display data, and counted the number of pattern matches found in *S. cerevisiae* proteins with at least one putative ortholog in the other ten yeast genomes considered in our study.

Using T-Coffee [38], an alignment of all putative orthologs (obtained using the BLAST reciprocal best hit method [39]) of *S. cerevisiae* proteins containing sequences matching a consensus sequence for an SH3 domain was carried out. This alignment was then used to determine the level of conservation of putative target ligand sites by searching for sequences matching the same consensus sequence in the orthologs. We did not search for conservation of putative target motifs in genomes without an ortholog for the domain under consideration. If there is no ortholog SH3 domain in the comparing species then the conservation of the motif in the ortholog of the putative target is not biologically relevant and should not be counted to increase our confidence in the putative interaction. Having said this, it should be noted that there could be several technical reasons why the ortholog of an SH3 domain could be missed in a genome. There might be errors in the genome assembly, genome annotations, domain annotation, or ortholog assignment. Thus, we also tried to calculate conservation scores without disregarding genomes with no ortholog for the domain under consideration. While this did not change the results significantly (data not shown), we felt that the first approach was more stringent.

In the orthologs, the search was restricted to a window surrounding the putative target ligand in the *S. cerevisiae* sequence, and we called this the probing region. In Figure 2, accuracy versus coverage for increasing probing regions is plotted, and it can be seen that by searching in a wider region of the alignment both coverage and accuracy are increased, especially for higher conservation scores (the complete analysis with the number of hits and false and true positives for each positive set is given in Table S1). Optimal results were obtained using a probing region of 210 alignment positions. It is important to emphasize that these were not necessarily amino acids, but 100 gaps or amino acids on each side of a motif of ten amino acids. This result could be due to poor alignment of some proteins, especially those rich in proline sequences. In fact most of the gain in coverage was due to interactions with proline-rich proteins that were difficult to align and had multiple gaps (i.e., Las17p, App1p, and Vrp1p). Also, these data may suggest that these small target ligands may be easily moved in primary sequence space during evolution, owing to compensatory mutations in proteins that are already proline-rich in nature. For both sets of positives a big improvement in accuracy was observed when we selected for consensus sequence conserved in the five genomes used (3.8-fold increase with the gold positives and 3.3-fold increase with the platinum positives). There was, however, a similar fold reduction in the coverage, 3-fold for the gold and 4.3-fold for the platinum set.

Since most known target proteins in the SH3 interaction network are proline-rich and a large probing window was used, it is possible that the hits found in orthologs were due to chance and lacked biological meaning. To eliminate this possibility two “decoy” proline-rich patterns were analyzed: PXXXPXXXP and EXXPXXP (where X is any amino acid), different from the consensus sequences. Both patterns were found with high frequency (>400 hits) on *S. cerevisiae* proteins. Using these two patterns, a loss in accuracy and coverage was observed (an average of 1.4 times less accuracy and 1.2 times less coverage for the PXXXPXXXP motif and an average of 3.4 times less accuracy and 2.5 times less coverage for the EXXPXXP motif). Thus, we can rule out the possibility that the results were generated by chance and can confirm that the observed phenomenon was the conservation of specific SH3 binding motifs and not of proline-rich tracks.

However, the accuracy obtained with conservation alone was still poor (using the gold set, accuracy = 46% and coverage = 31%, and using the platinum set, accuracy = 30% and coverage = 16%). A hypergeometric test allowed us to say that that the improvement in both positive sets and for all conservation scores was significant (*p* < 0.05) and not due to random sampling.

### Combining Comparative Genomics and Disorder Prediction

Since SH3 domains generally bind linear amino acid stretches, we tried to improve the accuracy of our consensus-based method by extracting secondary structure information about the sequences containing the target motifs. It has been argued that there might be biological advantages in presenting binding sites within unstructured regions [23–26]. It has also been observed that small linear motifs tend to accumulate in protein regions predicted to be intrinsically disordered [30] and that proline-rich regions are usually devoid of secondary structure [31]. To our knowledge there is no clear experimental evidence to support that SH3 domain target sites are generally unstructured before binding, but since SH3 domains bind small linear peptide motifs that are proline-rich, we hypothesized that SH3 domain targets might be mainly found in unstructured regions of the polypeptide chain. Therefore we used GlobPlot [30] in combination with coil-region predictions [40] to identify and study all consensus sequences found within disordered protein regions.

**Figure 3:**
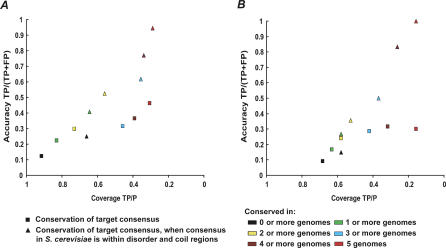
Combining Conservation and Secondary Structure Prediction We calculated, with the gold (A) and platinum (B) positive sets, the accuracy and coverage for target prediction when including or excluding secondary structure information. We used a probing region of 210 alignment positions in this analysis.

Combining disorder prediction with comparative genomics resulted in a significant (*p <* 0.01, using a hypergeometric test) increase in the accuracy of protein target prediction (there was a 2-fold average increase in both sets) (Figure 3). The decrease in coverage was 1.4-fold for the gold and 1.1-fold for the platinum set. For consensus sequence conserved in five or more genomes, we obtained 94% accuracy with 28% coverage for the gold set. For consensus sequence conserved in four or more genomes, we obtained 83% accuracy with 26% recovery for the platinum set. These results argue that intrinsic disorder plays an important role in SH3 protein interactions; however, further experimental work is needed to verify this observation.

Since the platinum positive set was independent (see Materials and Methods), the values obtained with this set may be used as a score for the performance of our method compared to others. Higher values of coverage and accuracy with the gold positive set were observed when using our method, but it should be noted that this could be due to a possible bias (see Materials and Methods). A detailed record of the number of hits and false and true positives for each conservation level in both positive sets can be found in Table S2.

Using the methods described in this work, we show proof of concept on how to integrate secondary structure prediction with comparative genomics to increase the accuracy of consensus-based prediction of peptide recognition modules. However, the method employed involves a clear trade-off between accuracy and coverage.

Of the 59 interactions in the final high-confidence interaction presented by Tong et al. [22], the method was able to predict 20 interactions when restricting for consensus sequences within disorder and found in four of the five genomes used. We tried to look for distinguishing features within these 20 interactions, compared to the remaining 39 that the method did not predict. There were no statistical differences in the average size of protein targets (*p =* 0.32 with a *t*-test), average proline content of protein targets (*p =* 0.12 with a *t*-test), usage of Class II motif (*p =* 0.21 with a hypergeometric distribution test), or conservation of SH3 domain (*p =* 0.82 with a *t-*test). There was a statistically significant difference in the average conservation of the target proteins (*p =* 0.03 with a *t*-test). The protein targets the method was able to predict were on average conserved in 8.7 of the ten species, while the targets not recovered were conserved in 7.6 species. This small but significant difference highlights the bias this method has for conserved interactions. A higher level of confidence can be placed in any putative target motif found conserved in most yeast species analyzed, but this level of conservation will only happen for essential interactions. It is important to note that for this reason this method will always miss species-specific protein interactions. However, adding more genomes of species within an appropriate divergence time should alleviate this problem, a concept discussed in more detail below. Another possible cause of loss in coverage could be interactions that are mediated by currently uncharacterized motifs or through noncanonical SH3 binding (i.e., through globular regions of the target protein).

As shown by other authors (reviewed in [41]), it should be possible to further improve the reliably of a protein interaction network, and therefore our method, by adding information from other sources of data (i.e., RNA expression, and essentiality and function information). This is especially true if the information is efficiently combined, e.g., employing a Bayesian network [12]. It was our intention to develop a method that could be used in species where these sources of information were not available, but in the future we will try to develop weighting schemes to include such sources for prediction of interactions mediated by small linear motifs.

### Determining an Optimal Divergence Time for the Genomes Used When Searching for Conservation of Target Ligands of SH3 Domains

**Figure 4:**
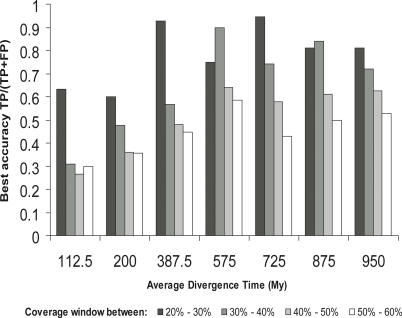
Optimal Divergence Time to Search for Conservation of Target Motif of SH3 Domains We designated seven groups of species with an increasing average divergence time from *S. cerevisiae* and calculated for each group the highest accuracy obtained for restricted windows of coverage. We used the gold positive and the negative set to calculate the accuracy and coverage (see Materials and Methods). The seven groups of species are as follows: (1) *S. bayanus, S. paradoxus, S. mikatae,* and *C. glabrata* (average divergence of 112.5 My from *S. cerevisiae*); (2) *S. paradoxus, S. mikatae, C. glabrata,* and *K. lactis* (average divergence of 200 My from *S. cerevisiae*); (3) *S. mikatae, C. glabrata, K. lactis,* and *C. albicans* (average divergence of 387.5 My from *S. cerevisiae*); (4) *C. glabrata, K. lactis, C. albicans,* and *D. hansenii* (average divergence of 575 My from *S. cerevisiae*); (5) *K. lactis, C. albicans, D. hansenii,* and *Y. lipolytica* (average divergence of 725 My from *S. cerevisiae*); (6) *C. albicans, D. hansenii, Y. lipolytica,* and *N. crassa* (average divergence of 875 My from *S. cerevisiae*); and (7) *D. hansenii, Y. lipolytica, N. crassa,* and *Sch. pombe* (average divergence of 950 My from *S. cerevisiae*). The individual values for the divergence time from *S. cerevisiae* were taken from the literature [32,42,43]. Although we tried to create groups that would not have genomes of species with very different separation dates from *S. cerevisiae,* it should be noted that because of the small number of available genomes, the groups are not homogenous. Also, the values of the divergence time of each species were not always obtained with the same method. Therefore, this range of values should be viewed critically.

Included in our initial hypothesis was the notion that there might be an optimal time of divergence to efficiently use the comparative genomics approach. To test this, phylogenetic data [32,42,43] with approximate values for the divergence times of the yeast species from *S. cerevisiae* (see Materials and Methods) were used to create seven groups of four genomes with increasing average divergence time from *S. cerevisiae*. Using the gold positives, the highest accuracy obtained for a small range of coverage values was determined for each of these groups. For different coverage ranges the highest accuracy was generally obtained with groups of genomes that had diverged from *S. cerevisiae* on average around 400–950 million years (My) (Figure 4).

**Figure 5:**
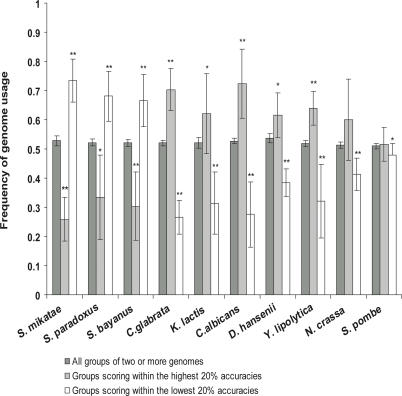
Most Informative Genomes in the Search for Conservation of Target Motif of SH3 Domains We created all possible combinations of two or more genomes of our set of ten genomes. For each combination we calculated the highest accuracy obtained for 11 windows of coverage from 15% to 70% at intervals of 5%. We then calculated the average frequency, over all coverage windows, of each individual species in all groups of genomes, in the combinations of genomes scoring within the 20% highest accuracy values and in the combinations scoring in the lowest 20% values of accuracy. We then used a *t*-test to determine, for each species, whether the average frequencies within the highest and lowest combinations were significantly different from the frequency in all possible combinations. *, *p <* 0.05; **, *p <* 0.001.

To explore this issue further we tried to find out which genomes might be more or less informative for our consensus-based predictions. For each possible combination of two or more genomes we calculated the highest accuracy obtained for 11 small windows of coverage (with intervals of 5% of coverage from 15% to 70%). Figure 5 shows the average of the individual genome representations in all possible groups, in the groups scoring in the highest 20% accuracies and in the groups scoring within the lowest 20% accuracies, over all the coverage windows studied. For each species, a *t*-test determination was carried out to see whether the average frequencies within the highest and lowest combinations were significantly different from the frequency in all possible combinations. From the analysis of the results the more informative genomes are *C. albicans, D. hansenii, C. glabrata,* *K. lactis,* and *Y. lypolytica*. We can also see that *N. crassa* and *Sch. pombe* are not over-represented in the highest scoring groups, suggesting that they have less informative genomes. More importantly, it is clear that including the genomes of *S. bayanus,* *S. mikatae,* or *S. paradoxus* leads to a decrease in the accuracy of predictions. These observations correlate well with the degree of divergence observed for the SH3 domains (see Figure 1) and with our proposed range for optimal divergence time.

In a recent report Eddy [44] used a theoretical model to study the statistical power of comparative genome sequence analysis. The model showed that, at close evolutionary distances, the number of comparative genomes needed to obtain the same statistical power increases. The model also suggests that the decline in statistical power for divergence times above optimal is smaller than for divergence times below optimal. In general our results support some of the proposals made by this model. According to the model it should be possible to obtain a high accuracy with closely divergent species but it would be necessary to use considerably more genomes at that distance. The author suggests that, for example, for human/baboon distances it would be necessary to use about seven times more genomes than at human/mouse distance to obtain the same statistical strength. For future work, we are therefore considering extending our method to include a weighing scheme based on the evolutionary distance between the comparing species and the target species. We think this could be achieved using an adaptation of the theoretical model proposed by Eddy [44].

It would be also interesting to study how many genomes would suffice to accurately predict an SH3 target interaction. Since the decrease in statistical power for *Sch. pombe* and *N. crassa* is small compared to species closely related to *S. cerevisiae,* we calculated the accuracy and coverage after addition of one or two of these species, to the five species selected previously, for different conservation scores. In general, an increase in coverage with little or no decrease in accuracy was observed (see Table S3). Addition of any of the closely related species, instead of *N. crassa* or *Sch. pombe,* resulted in a large loss of accuracy with moderate gain in coverage (results not shown). We believe that the improvement gained by adding species within the optimal divergence time would be better than that observed with *N. crassa* and *Sch. pombe*. The result generated with the latter two species suggests only that a sufficient number of genomes was not reached, since addition of more genomes still improved our scores. However, at present there are not enough genomes available to empirically tackle this question of a sufficient number of genomes for SH3 target prediction.

We believe the main factor determining the optimum divergence time is the conservation level of the biological feature. A biological feature that has higher conservation will require genomes of more divergent species to be accurately identified. Interaction types that are equally conserved should be accurately predicted with genomes of species at the same divergence times. This might mean that the same genomes could be used to predict interactions for other protein domains that bind small linear peptides (i.e., PDZ, WW, SH2, 14–3–3). Other interaction types that are mediated by larger interaction surfaces are probably more conserved and therefore might require genomes from more divergent species.

Although some results [34,35] have shown the importance of having genomes of recently divergent species in the study of DNA regulatory regions, recent findings [45] have shown that regulatory systems can be conserved over hundreds of millions of years. We argue that the concept of optimal divergence time presented should also be taken into consideration for protein–DNA interactions.

In this paper we show that for the study of SH3 protein interactions the genomes with more relevant information are from species that diverged around 400–950My ago from the species of interest. As was suggested by Eddy [44], this optimum might be specific for the particular interaction type being analyzed. Nevertheless, we believe that our results should be taken into consideration when identifying other biological features using comparative genome sequence analysis.

### Predictions of Novel SH3–Linear Peptide Interactions

**Figure 6:**
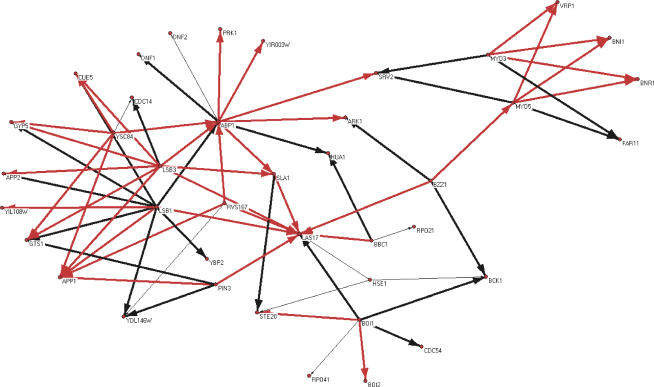
Predictions of *S. cerevisiae* SH3 Interactions We considered that a potential target consensus sequence, found by pattern matching, in an *S. cerevisiae* protein would be biologically relevant if it was within an unstructured region of the *S. cerevisiae* protein and also conserved in four of the seven comparison genomes used. *(C. glabrata, K. lactis, C. albicans, D. hansenii, Y. lipolytica, N. crassa,* and *Sch. pombe)*. Red lines indicate the interactions for which we found some experimental evidence in protein interaction databases [59–61]; thin black lines indicate interactions between proteins that are labeled as locating to different compartments; thick black lines indicate interactions for which we found no evidence. There were two *S. cerevisiae* SH3 domains for which we could not predict any interaction because of the stringency applied. A complete list of the interactions with function, localization, and binding positions is given in Table S4.

We used the method described above and the genomes of *C. glabrata, K. lactis, C. albicans, D. hansenii, Y. lipolytica, N. crassa,* and *Sch. pombe* to predict a set of 69 interactions regarding consensus sequence conserved in four of the seven genomes used (see Figure 6 and Table S4 for a complete list of the predicted interactions). Genomes of species that were over-represented in groups of genomes scoring within the 20% highest accuracies or under-represented in groups of genomes scoring within the 20% lowest accuracies were used. Some experimental evidence was found to support 37 of these interactions, all of which occurred between proteins labeled as belonging to the same compartments. Of the 32 remaining predictions, eight might not be possible since the putative interaction partners are annotated as having different cellular compartments, although in some cases a link between the two compartments could be possible (see below for some examples). Benchmarking with the gold positive and negative sets resulted in an accuracy of 73% and coverage of 37%. The level of conservation was chosen to allow for higher coverage, but it is important to note that higher accuracy for particular interactions can depend on the degree of conservation observed. We have included information about this in Table S4.

As expected we obtained a highly interconnected network with a very significant over-representation of proteins participating in processes typically associated with SH3 domains in *S. cerevisiae*. GO::TermFinder [46] was used to find significantly shared GO terms within the list of targets of the predictions. Amongst the most significant process associations found were cytoskeleton organization and biogenesis (*p =* 3.67 × 10^−15^), morphogenesis (*p =* 7.62 × 10^−12^), establishment of cell polarity (*p =* 1.19 × 10^−11^), actin cortical patch assembly (*p =* 5.09 × 10^−9^), and bud site selection (*p =* 1.28 × 10^−8^).

Some of the proposed interactions were further explored taking into account which *S. cerevisiae* biological processes these proteins were involved in. An interesting example is the proposed interaction between Abp1p with the P-type ATPases Dnf1p and Dnf2p. These proteins are required for phospholipid translocation and they mainly localize to the plasma membrane and intercellular compartments. The regulation of the lipid bilayer arrangement by Dnf1p and Dnf2p was demonstrated to be critical for budding endocytic vesicles [47]. It is also known that Abp1p is one of the activators of the Arp2/3 complex and is important in coupling the actin and membrane dynamics during endocytosis [48]. Following from the proposed interaction seen using our method, we suggest that Abp1p might target Dnf1p and Dnf2p to sites of endocytosis to play a role in endocytic vesicle formation or maintenance.

In order to calculate accuracy and coverage scores, we initially considered as “negative” interactions between proteins that did not share the same cellular compartment. After having obtained our list of predicted interactions, we decided to investigate them without disregarding these “negative” interactions. This decision was made because the negative set is based in part on high-throughput measurements that do not take into account the dynamics of cellular localization. Two proteins might not share a compartment in a given cellular condition, but this might change in different cellular states (examples in *S. cerevisiae* include cell cycle, pheromone response, and filamentous growth). This reasoning actually leads us to think that the localization data on proteins are underevaluated and, if anything, will result in an underestimation of our accuracy scores.

Within our set of final predictions, Hse1p-mediated interactions are examples of those occurring between proteins marked as belonging to different compartments. According to our results the SH3 domain of Hse1p has a high probability of binding to proline-rich regions of Ste20p, Bck1p, and Las17p. Hse1p was recently reported to be part of a complex that binds ubiquitin and is important in sorting proteins in the endosome [49,50]. Knowing that both Ste20p and Bck1p are involved in the response to mating and that Hse1p is involved in the trafficking/sorting of the alpha-factor pheromone receptor, these SH3 domain interactions might be part of the sorting mechanism of the alpha receptor in the multivesicular bodies. Activated alpha-factor pheromone receptors recruit Ste20p by the dissociation of Gβγ subunits (reviewed in [51]). There is some evidence that Ste20p activation can lead to the phosphorylation of Bck1p in the mating response [52]. Activated mating receptors are internalized after phosphorylation and ubiquitination of their carboxy-terminal tails and are targeted to the vacuole for degradation [53]. We propose that these internalized vesicles are decorated with complexes containing Ste20p, Bck1p, and Las17p and that the interaction of the SH3 domains of Hse1p with these proteins might be important in the sorting of internalized mating receptors.

### Conclusion

We present here a method to predict biologically relevant protein interactions mediated by peptide recognition modules. Conservation of target linear peptides and analysis of protein disorder can be effectively combined to screen for biologically relevant interactions that are predicted from binding matrixes obtained from experimental data. However, the method has a small coverage and still relies on experimental determination of the SH3 target consensus sequence. In the future it should be possible to predict the target motifs using available structural data and homology modeling [54,55].

This study provides some evidence for the importance of intrinsic disorder in the context of protein interactions. Specifically, binding motifs within disordered protein regions are more likely to be biologically relevant binding sites than equivalent sites within ordered regions. To our knowledge there is no experimental evidence currently available to support the idea that in general SH3 domains bind within unstructured regions; therefore, particular cases should be investigated carefully. Nevertheless, we hope our observations will contribute to discussion of the role of intrinsically disordered protein regions.

The analysis carried out demonstrated that there is an optimal divergence time for the species to be included in comparative genomics when looking for the conservation of binding sites of peptide recognition modules. For SH3 domains in yeast, this interval is between 400 and 950 My, and although these divergence times may be specific to SH3 domains and to yeast evolution, the concept should be taken into consideration for future comparative studies.

Finally we have used this method to predict novel SH3–linear peptide interactions for *S. cerevisiae*. The interaction map obtained contains information on the binding regions of both interaction partners and should allow experimentalists to devise effective and precise system perturbations by targeting a particular interaction.

## Materials and Methods

### SH3 domain conservation.

We created a phylogenetic tree (see Dataset S1) produced by the neighbor-joining method from a ClustalW alignment [56] of the SH3 domains of the 13 yeast species in our set. The SH3 domains were identified using SMART [57]. Putative orthologs for all *S. cerevisiae* proteins were determined by the BLAST reciprocal best hit method [39]. We considered that a putative ortholog of a S*. cerevisiae* SH3 domain was not conserved if the two domains were not in the same branch of the phylogenetic tree.

After eliminating these “divergent” domains, we did multiple sequence alignments of the groups of orthologous domains. To determine the binding positions, we included in the alignments the SH3 domain of Fyn. From visual inspection of crystal structures of complexes of SH3 domains with ligands, we decided to analyze the positions Tyr91, Tyr93, Arg96, Thr97, Asp99, Asp100, Asp118, Trp119, Tyr132, Pro134, and Tyr137 of Fyn that we considered might influence binding specificity. By manual inspection of the alignments we extracted the positions of all domains corresponding to the positions of the Fyn SH3 domain that are important for binding specificity and determined their conservation. Any substitution that scored a non-negative value in the blosum62 matrix that would not result in a reversal of charge was considered to be conserved.

### Positive and negative datasets.

We considered a positive set of 59 interactions (containing 15 different SH3 domains from 15 different proteins) defined by Tong et al. [22]; this we called the gold set. Tong et al. obtained the final set of interactions by the overlap of two sets of interactions obtained with two different methods. They used phage display data to create a PSSM and used it to scan the *S. cerevisiae* proteome. Using a threshold on the PSSM they selected the first set of interactions, then they created a second interaction network by yeast two-hybrid screening and obtained the final network (our gold set) by the overlap of the two.

We considered a second positive standard, which we called the platinum set, of higher confidence, with 19 interactions (containing ten different SH3 domains from ten different proteins) derived from the overlap of the two-hybrid assays, obtained from Tong et al. [22], with the MIPS complexes dataset [58].

The two positive datasets overlap only partially (ten interactions from the platinum set are also in the gold set).

To build our negative dataset we assumed that two proteins that do not share the same subcellular compartment according to MIPS localization data [58] cannot interact, and we compiled a list of all *S. cerevisiae* proteins pairs that do not share at least one subcellular compartment.

Since we also used the phage display data from Tong et al. [22] to derive the consensus sequences recognized by the yeast SH3s used in this study, the gold set might be biased. We would like to stress that we did not use a PSSM as in the Tong et al. paper and therefore even our initial motif-based predictions without any filtering are not the same as the network obtained by Tong et al. with the phage display data.

We did not merge the two positive datasets, thus keeping the platinum one as a truly independent positive dataset. We decided to also use the gold set because although it is not appropriate to use the absolute performance value calculated with this set to compare our method with others, it still served as a check for the relative performance of different filters of our method.

### Accuracy and coverage determination.

The ratio between true positives (TP) and the sum of true positives plus false positives (FP) was used as a measure of accuracy. True positives were the number of predicted interactions within a positive set. False positives were the number of predicted interactions found within the negative set. To measure the coverage of the methods, we tracked the ratio TP/P, where P is the total number of positives in the positive set.

### Estimated divergence time from *S. cerevisiae.*

The estimated divergence times of the other yeast species from *S. cerevisiae* were as follows: *C. glabrata,* 300 My; *D. hansenii,* 800 My; *K. lactis,* 400 My; *Y. lipolytica,* 900 My; *C. albicans,* 800 My; *S. paradoxus,* 50 My; *S. bayanus,* 50 My; *S. mikatae,* 50 My; *N. crassa,* 1,000 My; and *Sch. pombe,* 1,100 My. These values were based on phylogenetic studies found in the literature [32,42,43].

## Supporting Information

Dataset S1 Phylogenetic Tree of the SH3 Domains in the Study The phylogenetic tree of all the SH3 domains of the yeast species in our study.
(14 KB DND)

Table S1 Detailed Analysis of the Conservation of Target Consensus Sequence in Putative Targets of *S. cerevisiae* SH3 Domains (248 KB PDF)

Table S2 Detailed Analysis of the Conservation of Target Consensus Sequence in Putative Targets of *S. cerevisiae* SH3 Domains within Unstructured Regions of Proteins (248 KB PDF)

Table S3 Effect of Addition of More Informative Genomes on Accuracy and Coverage Scores (17 KB PDF)

Table S4 List of Predicted Interactions (52 KB PDF)

## Abbreviations

My: million years
PSSM: position-specific scoring matrix
