# Supplementary material for: Comparative Genomics and Disorder Prediction Identify Biologically Relevant SH3 Protein Interactions
Source: PLoS Comput Biol. 2005 Aug 12;1(3):e26. doi: 10.1371/journal.pcbi.0010026 (PMC1187863; doi:10.1371/journal.pcbi.0010026)
Supplement: Supplementary file 1 [file pcbi.0010026.st001.pdf]

Supplementary Table I – **Detailed analysis of the conservation of target consensus on putative targets of *S. cerevisiae* SH3 domains.** We used the SH3 domain binding motifs, extracted from Tong *et al*. to scan the S. cerevisiae proteome for predicted binding sites. We then determined how many of these sites are conserved in ortholog proteins using different sizes for the probing region. It is important to mention that the size of the probing region includes gaps and amino acids and therefore the found hits could be much closer to the region of the ortholog protein aligned with the *S. cerevisiae* target. We calculated also the accuracy (TP/(TP+FP)) and coverage (TP/P) with both positive datasets.

Size of probing region	Conservation of target motifs of S. cerevisiae SH3 domains										
	Calculated with Gold Positives (Total Positives =59)						Calculated with Platinum Positives (Total Positives =19)				
30	Cons. Score	Total	TP	FP	TP/(TP+FP)	TP/P	Total	TP	FP	TP/(TP+FP)	TP/P
	0	1148	54	390	0.1216216	0.9152542	476	13	131	0.0902778	0.6842105
	1	490	48	158	0.2330097	0.8135593	210	11	58	0.1594203	0.5789474
	2	309	33	91	0.266129	0.559322	136	7	32	0.1794872	0.3684211
	3	194	23	55	0.2948718	0.3898305	88	7	17	0.2916667	0.3684211
	4	136	18	37	0.3272727	0.3050847	66	5	12	0.2941176	0.2631579
	5	67	9	21	0.3	0.1525424	37	3	7	0.3	0.1578947
70	Cons. Score	Total	TP	FP	TP/(TP+FP)	TP/P	Total	TP	FP	TP/(TP+FP)	TP/P
	0	1148	54	390	0.1216216	0.9152542	476	13	131	0.0902778	0.6842105
	1	502	48	159	0.2318841	0.8135593	213	11	58	0.1594203	0.5789474
	2	318	38	93	0.2900763	0.6440678	141	7	34	0.1707317	0.3684211
	3	202	27	55	0.3292683	0.4576271	92	7	17	0.2916667	0.3684211
	4	140	20	37	0.3508772	0.3389831	69	5	12	0.2941176	0.2631579
	5	68	9	21	0.3	0.1525424	37	3	7	0.3	0.1578947
110	Cons. Score	Total	TP	FP	TP/(TP+FP)	TP/P	Total	TP	FP	TP/(TP+FP)	TP/P
	0	1148	54	390	0.1216216	0.9152542	476	13	131	0.0902778	0.6842105
	1	515	49	164	0.2300469	0.8305085	216	12	58	0.1714286	0.6315789
	2	327	40	96	0.2941176	0.6779661	144	9	34	0.2093023	0.4736842
	3	207	27	57	0.3214286	0.4576271	94	7	18	0.28	0.3684211
	4	142	22	37	0.3728814	0.3728814	70	6	12	0.3333333	0.3157895
	5	75	15	21	0.4166667	0.2542373	42	3	7	0.3	0.1578947
210	Cons. Score	Total	TP	FP	TP/(TP+FP)	TP/P	Total	TP	FP	TP/(TP+FP)	TP/P
	0	1148	54	390	0.1216216	0.9152542	476	13	131	0.0902778	0.6842105
	1	533	49	170	0.2237443	0.8305085	220	12	60	0.1666667	0.6315789
	2	349	43	101	0.2986111	0.7288136	154	11	35	0.2391304	0.5789474
	3	214	27	59	0.3139535	0.4576271	97	8	20	0.2857143	0.4210526
	4	149	23	40	0.3650794	0.3898305	73	6	13	0.3157895	0.3157895
	5	80	18	21	0.4615385	0.3050847	44	3	7	0.3	0.1578947
410	Cons. Score	Total	TP	FP	TP/(TP+FP)	TP/P	Total	TP	FP	TP/(TP+FP)	TP/P
	0	1148	54	390	0.1216216	0.9152542	476	13	131	0.0902778	0.6842105
	1	556	50	175	0.2222222	0.8474576	230	12	61	0.1643836	0.6315789
	2	363	44	104	0.2972973	0.7457627	156	11	35	0.2391304	0.5789474
	3	230	31	62	0.3333333	0.5254237	106	8	21	0.2758621	0.4210526
	4	153	26	42	0.3823529	0.440678	75	6	14	0.3	0.3157895
	5	89	20	24	0.4545455	0.3389831	46	3	7	0.3	0.1578947
610	Cons. Score	Total	TP	FP	TP/(TP+FP)	TP/P	Total	TP	FP	TP/(TP+FP)	TP/P
	0	1148	54	390	0.1216216	0.9152542	476	13	131	0.0902778	0.6842105
	1	569	50	181	0.2164502	0.8474576	232	12	62	0.1621622	0.6315789
	2	373	44	107	0.2913907	0.7457627	158	11	35	0.2391304	0.5789474
	3	237	31	63	0.3297872	0.5254237	108	8	21	0.2758621	0.4210526
	4	159	27	43	0.3857143	0.4576271	77	7	14	0.3333333	0.3684211
	5	93	21	26	0.4468085	0.3559322	47	3	7	0.3	0.1578947
