# Supplementary material for: Comparative Genomics and Disorder Prediction Identify Biologically Relevant SH3 Protein Interactions
Source: PLoS Comput Biol. 2005 Aug 12;1(3):e26. doi: 10.1371/journal.pcbi.0010026 (PMC1187863; doi:10.1371/journal.pcbi.0010026)
Supplement: Supplementary file 2 [file pcbi.0010026.st002.pdf]

Supplementary Table II – **Detailed analysis of the conservation of target consensus on putative targets of *S. cerevisiae* SH3 domains, within unstructured regions of proteins.** We used the SH3 domain binding motifs, extracted from Tong *et al*. to scan the *S. cerevisiae* proteome for predicted binding sites that were within unstructured parts of proteins of the putative targets. We then determined how many of these sites are conserved in ortholog proteins using different sizes for the probing region. It is important to mention that the size of the probing region includes gaps and amino acids and therefore the found hits could be much closer to the region of the ortholog protein aligned with the *S. cerevisiae* target. We calculated also the accuracy (TP/(TP+FP)) and coverage (TP/P) with both positive datasets.

Size of probing region	Conservation of target motifs of S. cerevisiae SH3 domains										
	Calculated with Gold Positives (Total Positives =59)						Calculated with Platinum Positives (Total Positives =19)				
30	Cons. Score	Total	TP	FP	TP/(TP+FP)	TP/P	Total	TP	FP	TP/(TP+FP)	TP/P
	0	395	39	117	0.25	0.6610169	221	11	63	0.1486486	0.5789474
	1	180	36	48	0.4285714	0.6101695	105	10	29	0.2564103	0.5263158
	2	111	25	24	0.5102041	0.4237288	69	6	14	0.3	0.3157895
	3	73	20	11	0.6451613	0.3389831	46	6	5	0.5454545	0.3157895
	4	50	16	5	0.7619048	0.2711864	31	4	1	0.8	0.2105263
	5	18	9	1	0.9	0.1525424	12	3	0	1	0.1578947
70	Cons. Score	Total	TP	FP	TP/(TP+FP)	TP/P	Total	TP	FP	TP/(TP+FP)	TP/P
	0	395	39	117	0.25	0.6610169	221	11	63	0.1486486	0.5789474
	1	188	37	49	0.4302326	0.6271186	107	10	29	0.2564103	0.5263158
	2	119	29	27	0.5178571	0.4915254	75	6	17	0.2608696	0.3157895
	3	78	21	11	0.65625	0.3559322	48	6	5	0.5454545	0.3157895
	4	53	17	5	0.7727273	0.2881356	33	4	1	0.8	0.2105263
	5	19	9	1	0.9	0.1525424	12	3	0	1	0.1578947
110	Cons. Score	Total	TP	FP	TP/(TP+FP)	TP/P	Total	TP	FP	TP/(TP+FP)	TP/P
	0	395	39	117	0.25	0.6610169	221	11	63	0.1486486	0.5789474
	1	197	38	52	0.4222222	0.6440678	109	11	29	0.275	0.5789474
	2	123	30	27	0.5263158	0.5084746	78	8	17	0.32	0.4210526
	3	80	21	12	0.6363636	0.3559322	50	6	6	0.5	0.3157895
	4	55	19	5	0.7916667	0.3220339	34	5	1	0.8333333	0.2631579
	5	23	13	1	0.9285714	0.220339	15	3	0	1	0.1578947
210	Cons. Score	Total	TP	FP	TP/(TP+FP)	TP/P	Total	TP	FP	TP/(TP+FP)	TP/P
	0	395	39	117	0.25	0.6610169	221	11	63	0.1486486	0.5789474
	1	208	38	55	0.4086022	0.6440678	110	11	30	0.2682927	0.5789474
	2	136	33	30	0.5238095	0.559322	82	10	18	0.3571429	0.5263158
	3	86	21	13	0.6176471	0.3559322	52	7	7	0.5	0.3684211
	4	61	20	6	0.7692308	0.3389831	38	5	1	0.8333333	0.2631579
	5	30	17	1	0.9444444	0.2881356	20	3	0	1	0.1578947
410	Cons. Score	Total	TP	FP	TP/(TP+FP)	TP/P	Total	TP	FP	TP/(TP+FP)	TP/P
	0	395	39	117	0.25	0.6610169	221	11	63	0.1486486	0.5789474
	1	217	37	58	0.3894737	0.6271186	117	11	31	0.2619048	0.5789474
	2	144	32	32	0.5	0.5423729	85	10	18	0.3571429	0.5263158
	3	92	23	15	0.6052632	0.3898305	56	7	8	0.4666667	0.3684211
	4	64	22	8	0.7333333	0.3728814	40	5	2	0.7142857	0.2631579
	5	37	18	3	0.8571429	0.3050847	22	3	0	1	0.1578947
610	Cons. Score	Total	TP	FP	TP/(TP+FP)	TP/P	Total	TP	FP	TP/(TP+FP)	TP/P
	0	395	39	117	0.25	0.6610169	221	11	63	0.1486486	0.5789474
	1	219	37	59	0.3854167	0.6271186	118	11	32	0.255814	0.5789474
	2	146	32	33	0.4923077	0.5423729	85	10	18	0.3571429	0.5263158
	3	99	23	16	0.5897436	0.3898305	58	7	8	0.4666667	0.3684211
	4	70	23	9	0.71875	0.3898305	42	6	2	0.75	0.3157895
	5	40	19	4	0.826087	0.3220339	23	3	0	1	0.1578947
