# Supplementary material for: Comparative Genomics and Disorder Prediction Identify Biologically Relevant SH3 Protein Interactions
Source: PLoS Comput Biol. 2005 Aug 12;1(3):e26. doi: 10.1371/journal.pcbi.0010026 (PMC1187863; doi:10.1371/journal.pcbi.0010026)
Supplement: Supplementary file 3 [file pcbi.0010026.st003.pdf]

Supplementary table III - **Effect of addition of more informative genomes on accuracy and coverage scores**. We calculated the accuracy and coverage after addition of *N. crassa* and *S. pombe* genomes, for different conservation scores. The analysis is only for putative target motifs within predicted unstructured protein regions in the *S. cerevisiae* proteome and for a probing region of 210.

	Calculated with Gold Positives (Total Positives =59)					Calculated with Platinum Positives (Total Positives =19)				
	Total	TP	FP	TP/(TP+FP)	TP/P	Total	TP	FP	TP/(TP+FP)	TP/P
Conserved in 1 of 5 genomes	208	38	55	0.41	0.64	110	11	30	0.27	0.58
Conserved in 1 of 6 genomes	215	38	58	0.40	0.64	115	11	31	0.26	0.58
Conserved in 1 of 7 genomes	216	38	58	0.40	0.64	115	11	31	0.26	0.58
Conserved in 2 of 5 genomes	136	33	30	0.52	0.56	82	10	18	0.36	0.53
Conserved in 2 of 6 genomes	140	34	31	0.52	0.58	85	11	18	0.38	0.58
Conserved in 2 of 7 genomes	145	34	34	0.50	0.58	87	11	20	0.35	0.58
Conserved in 3 of 5 genomes	86	21	13	0.62	0.36	52	7	7	0.50	0.37
Conserved in 3 of 6 genomes	89	23	13	0.64	0.39	54	8	7	0.53	0.42
Conserved in 3 of 7 genomes	92	24	13	0.65	0.41	56	9	7	0.56	0.47
Conserved in 4 of 5 genomes	61	20	6	0.77	0.34	38	5	2	0.71	0.26
Conserved in 4 of 6 genomes	67	21	8	0.72	0.36	43	6	3	0.67	0.32
Conserved in 4 of 7 genomes	69	22	8	0.73	0.37	43	6	3	0.67	0.32
Conserved in 5 of 5 genomes	30	17	1	0.94	0.29	20	3	0	1	0.16
Conserved in 5 of 6 genomes	40	20	3	0.87	0.34	28	5	0	1	0.26
Conserved in 5 of 7 genomes	45	20	4	0.83	0.34	30	5	0	1	0.26
