# Supplementary material for: Comparative Genomics and Disorder Prediction Identify Biologically Relevant SH3 Protein Interactions
Source: PLoS Comput Biol. 2005 Aug 12;1(3):e26. doi: 10.1371/journal.pcbi.0010026 (PMC1187863; doi:10.1371/journal.pcbi.0010026)
Supplement: Supplementary file 4 [file pcbi.0010026.st004.pdf]

Supplementary table IV – **List of predicted interactions**. We considered that a potential target consensus, found by pattern matching, in an *S. cerevisiae* protein would be biologically relevant if it was within an unstructured region of the *S. cerevisiae* protein and also conserved in four of the six genomes used. We used the genomes of *C. glabrata*, *K.lactis*, *C. albicans*, *D. hansenii*, *Y. lipolytica* and *N. crassa* because these species diverged from *S. cerevisiae* at a time that falls within the optimal range proposed in our analysis. We retrieved function and localization information from MIPS (Mewes, Frishman et al. 2000) and SGD databases.

SH3 protein	Localization	Function	Target protein	Localization	Function	Target binding positions	Accuracy %
LSB1	cytoplasm nucleus	biological_process unknown;intracellular signalling;	ABP1	cytoplasm actin cytoskeleton	cytoskeleton organization and biogenesis;assembly of protein complexes;protein binding;cell growth ;actin cytoskeleton;budding;	446-456	83%
LSB1	cytoplasm nucleus	biological_process unknown;intracellular signalling;	APP1	cytoplasm actin cytoskeleton nucleus	cytoskeleton organization and biogenesis;cell growth ;actin cytoskeleton;budding;	505-515	83%
LSB1	cytoplasm nucleus	biological_process unknown;intracellular signalling;	YBP2	cytoplasm	-	537-547	
LSB1	cytoplasm nucleus	biological_process unknown;intracellular signalling;	LAS17	cytoplasm actin cytoskeleton	cytokinesis; ;assembly of protein complexes;protein binding;endocytosis;osmotic and salt stress response;cell growth ;actin cytoskeleton;budding;	311-321<br>323-333<br>352-362<br>384-394	83%
LSB1	cytoplasm nucleus	biological_process unknown;intracellular signalling;	CUE5	cytoplasm	-	337-347	73%
LSB1	cytoplasm nucleus	biological_process unknown;intracellular signalling;	GYP5	bud neck cell periphery cytoplasm	vesicle-mediated transport;GTPase activator ;vacuolar transport;	279-289<br>293-303	83%
LSB1	cytoplasm nucleus	biological_process unknown;intracellular signalling;	APP2	bud neck cytoplasm mitochondria	cytoskeleton organization and biogenesis;actin cytoskeleton;	200-210	83%
LSB1	cytoplasm nucleus	biological_process unknown;intracellular signalling;	GTS1	cytoplasm actin cytoskeleton nucleus	morphogenesis;mitotic cell cycle and cell cycle control;transcriptional activator;drug transport;stress response;rhythm ;cell growth ;cell aging;budding;development of asco;	247-257	73%
LSB1	cytoplasm nucleus	biological_process unknown;intracellular signalling;	YIL108W	cytoplasm	-	577-587	73%
LSB1	cytoplasm nucleus	biological_process unknown;intracellular signalling;	YDL146W	bud neck cell periphery cytoplasm nucleus	-	477-487	73%
MYO5	actin cytoskeleton	cell wall organization and biogenesis;conversion to kinetic energy ;cytoskeleton;exocytosis;endocytosis;osmotic and salt stress response;osmosensing;cell wall;budding;	SRV2	cell periphery actin cytoskeleton	cytoskeleton organization and biogenesis;metabolism of cyclic and unusual nucleotides;protein binding;small GTPase mediated signal transduction;cell growth ;cytoskeleton;budding;	276-286	83%
MYO5	actin cytoskeleton	cell wall organization and biogenesis;conversion to kinetic energy ;cytoskeleton;exocytosis;endocytosis;osmotic and salt stress response;osmosensing;cell wall;budding;	FAR11	ambiguous	cell cycle;conjugation;cell cycle arrest;pheromone response;	520-530	73%
MYO5	actin cytoskeleton	cell wall organization and biogenesis;conversion to kinetic energy ;cytoskeleton;exocytosis;endocytosis;osmotic and salt stress response;osmosensing;cell wall;budding;	BNI1	neck neck cytoplasm cytoskeleton	response to stress;cytokinesis ;spindle pole body;protein binding;structural protein;RNA transport;small GTPase mediated signal transduction;osmotic and salt stress response;pheromone response;cell growth ;actin cytoskeleton;budding;	1238-1248<br>1277-1287	83%
MYO5	actin	cell wall organization and	VRP1	cytoplasm actin	vesicle-mediated transport;response	4-14	83%

	cytoskeleton	biogenesis;conversion to kinetic energy ;cytoskeleton;exocytosis;endocytosis;osmotic and salt stress response;osmosensing;cell wall;budding;		cytoskeleton	to stress;budding;protein binding;endocytosis;osmotic and salt stress response;cell growth ;actin cytoskeleton;	234-244 395-405	
MYO5	actin cytoskeleton	cell wall organization and biogenesis;conversion to kinetic energy ;cytoskeleton;exocytosis;endocytosis;osmotic and salt stress response;osmosensing;cell wall;budding;	BNR1	neck cell periphery actin cytoskeleton	response to stress;budding;protein binding;osmotic and salt stress response;cell growth ;actin cytoskeleton;	765-775 819-829	83%
YSC84	cytoplasm cytoskeleton	vesicle-mediated transport;endocytosis;cell growth ;actin cytoskeleton;budding;	ABP1	cytoplasm actin cytoskeleton	cytoskeleton organization and biogenesis;assembly of protein complexes;protein binding;cell growth ;actin cytoskeleton;budding;	447-457	83%
YSC84	cytoplasm cytoskeleton	vesicle-mediated transport;endocytosis;cell growth ;actin cytoskeleton;budding;	APP1	cytoplasm actin cytoskeleton nucleus	cytoskeleton organization and biogenesis;cell growth ;actin cytoskeleton;budding;	506-516	83%
YSC84	cytoplasm cytoskeleton	vesicle-mediated transport;endocytosis;cell growth ;actin cytoskeleton;budding;	CDC14	nucleus nucleolus	phosphate utilization;DNA synthesis and replication;mitotic cell cycle and cell cycle control;modification by phosphorylation;	121-131	73%
YSC84	cytoplasm cytoskeleton	vesicle-mediated transport;endocytosis;cell growth ;actin cytoskeleton;budding;	GTS1	cytoplasm actin cytoskeleton nucleus	morphogenesis;mitotic cell cycle and cell cycle control;transcriptional activator;drug transport;stress response;rhythm ;cell growth ;cell aging;budding;development of asco;	248-258	73%
YSC84	cytoplasm cytoskeleton	vesicle-mediated transport;endocytosis;cell growth ;actin cytoskeleton;budding;	CUE5	cytoplasm	-	338-348	83%
YSC84	cytoplasm cytoskeleton	vesicle-mediated transport;endocytosis;cell growth ;actin cytoskeleton;budding;	GYP5	bud neck cell periphery cytoplasm	vesicle-mediated transport;GTPase activator ;vacuolar transport;	280-290 294-304	83%
HSE1	endosome	transport;protein targeting;protein binding;vacuolar transport;	BCK1	cytoplasm	phosphate utilization;mitotic cell cycle and cell cycle control;modification by phosphorylation;MAPKKK cascade;stress response;perception of nutrients and nutritional adaptation;directional cell growth ;budding;	803-813	83%
HSE1	endosome	transport;protein targeting;protein binding;vacuolar transport;	LAS17	cytoplasm actin cytoskeleton	cytokinesis;assembly of protein complexes;protein binding;endocytosis;osmotic and salt stress response;cell growth ;actin cytoskeleton;budding;	308-318 319-329	83%
HSE1	endosome	transport;protein targeting;protein binding;vacuolar transport;	STE20	bud cytoplasm	pseudohyphal growth;phosphate utilization;modification by phosphorylation;transmembrane signal transduction;pheromone response;cell growth ;cytoskeleton;budding;	471-481	83%
ABP1	cytoplasm actin cytoskeleton	cytoskeleton organization and biogenesis;assembly of protein complexes;protein binding;cell growth ;actin cytoskeleton;budding;	SRV2	cell periphery actin cytoskeleton	cytoskeleton organization and biogenesis;metabolism of cyclic and unusual nucleotides;protein binding;small GTPase mediated signal transduction;cell growth ;budding;	351-361	83%
ABP1	cytoplasm actin cytoskeleton	cytoskeleton organization and biogenesis;assembly of protein complexes;protein binding;cell growth ;actin cytoskeleton;budding;	DNF2	bud cell periphery plasma membrane	peptide transport;protein transport;lipid transport;transport ATPases;	1468-1478	73%
ABP1	cytoplasm actin cytoskeleton	cytoskeleton organization and biogenesis;assembly of protein complexes;protein binding;cell growth ;actin cytoskeleton;budding;	PRK1	cytoplasm actin cytoskeleton	cytokinesis;phosphate utilization;mitotic cell cycle and cell cycle control;modification by phosphorylation;assembly of protein complexes;endocytosis;actin cytoskeleton;budding;	746-756	73%

ABP1	cytoplasm actin cytoskeleton	cytoskeleton organization and biogenesis;assembly of protein complexes;protein binding;cell growth ;actin cytoskeleton;budding;	HUA1	cytoplasm nucleus	cytoskeleton organization and biogenesis;assembly of protein complexes;cell growth ;actin cytoskeleton;budding;	70-80	83%
ABP1	cytoplasm actin cytoskeleton	cytoskeleton organization and biogenesis;assembly of protein complexes;protein binding;cell growth ;actin cytoskeleton;budding;	YIR003W	cytoplasm actin cytoskeleton	-	3-13<br>386-396	73%
ABP1	cytoplasm actin cytoskeleton	cytoskeleton organization and biogenesis;assembly of protein complexes;protein binding;cell growth ;actin cytoskeleton;budding;	ARK1	cytoplasm actin cytoskeleton	cytokinesis;phosphate utilization;modification by phosphorylation;assembly of protein complexes;endocytosis;actin cytoskeleton;budding;	605-615<br>616-626	83%
ABP1	cytoplasm actin cytoskeleton	cytoskeleton organization and biogenesis;assembly of protein complexes;protein binding;cell growth ;actin cytoskeleton;budding;	DNF1	plasma membrane cytoplasm golgi mitochondria	peptide transport;protein transport;lipid transport;transport ATPases;	1425-1435	73%
ABP1	cytoplasm actin cytoskeleton	cytoskeleton organization and biogenesis;assembly of protein complexes;protein binding;cell growth ;actin cytoskeleton;budding;	SLA1	cytoplasm actin cytoskeleton nucleus	cell wall organization and biogenesis;assembly of protein complexes;protein binding;endocytosis;cell growth ;cell wall;actin cytoskeleton;budding;	191-201	83%
BBC1	cytoplasm	cytoskeleton organization and biogenesis;protein binding;actin cytoskeleton;	HUA1	cytoplasm nucleus	cytoskeleton organization and biogenesis;assembly of protein complexes;cell growth ;actin cytoskeleton;budding;	73-83	73%
BBC1	cytoplasm	cytoskeleton organization and biogenesis;protein binding;actin cytoskeleton;	RPO21	nucleus nucleus mitochondria	transcription;general transcription activities;DNA binding;	241-251	83%
BBC1	cytoplasm	cytoskeleton organization and biogenesis;protein binding;actin cytoskeleton;	LAS17	cytoplasm actin cytoskeleton	cytokinesis;assembly of protein complexes;protein binding;endocytosis;osmotic and salt stress response;cell growth ;actin cytoskeleton;budding;	346-356<br>379-389	73%
LSB3	neck cell periphery cytoplasm cytoskeleton mitochondria	cytoskeleton organization and biogenesis;actin cytoskeleton;	ABP1	cytoplasm actin cytoskeleton	cytoskeleton organization and biogenesis;assembly of protein complexes;protein binding;cell growth ;actin cytoskeleton;budding;	446-456	83%
LSB3	neck cell periphery cytoplasm cytoskeleton mitochondria	cytoskeleton organization and biogenesis;actin cytoskeleton;	APP1	cytoplasm actin cytoskeleton nucleus	cytoskeleton organization and biogenesis;cell growth ;actin cytoskeleton;budding;	505-515	83%
LSB3	neck cell periphery cytoplasm cytoskeleton mitochondria	cytoskeleton organization and biogenesis;actin cytoskeleton;	LAS17	cytoplasm actin cytoskeleton	cytokinesis;assembly of protein complexes;protein binding;endocytosis;osmotic and salt stress response;cell growth ;actin cytoskeleton;budding;	311-321	83%
LSB3	neck cell periphery cytoplasm cytoskeleton mitochondria	cytoskeleton organization and biogenesis;actin cytoskeleton;	CUE5	cytoplasm	-	337-347	83%
LSB3	neck cell periphery cytoplasm cytoskeleton mitochondria	cytoskeleton organization and biogenesis;actin cytoskeleton;	GYP5	bud neck cell periphery cytoplasm	vesicle-mediated transport;GTPase activator ;vacuolar transport;	293-303	83%
LSB3	neck cell periphery cytoplasm cytoskeleton mitochondria	cytoskeleton organization and biogenesis;actin cytoskeleton;	APP2	bud neck cytoplasm mitochondria	cytoskeleton organization and biogenesis;actin cytoskeleton;	200-210	83%
LSB3	neck cell periphery cytoplasm cytoskeleton mitochondria	cytoskeleton organization and biogenesis;actin cytoskeleton;	CDC14	nucleus nucleolus	phosphate utilization;DNA synthesis and replication;mitotic cell cycle and cell cycle control;modification by phosphorylation;	120-130	73%

LSB3	neck cell periphery cytoplasm cytoskeleton mitochondria	cytoskeleton organization and biogenesis;actin cytoskeleton;	GTS1	cytoplasm actin cytoskeleton nucleus	morphogenesis;mitotic cell cycle and cell cycle control;transcriptional activator;drug transport;stress response;rhythm ;cell growth ;cell aging;budding;development of asco;	247-257	73%
LSB3	neck cell periphery cytoplasm cytoskeleton mitochondria	cytoskeleton organization and biogenesis;actin cytoskeleton;	SLA1	cytoplasm actin cytoskeleton nucleus	cell wall organization and biogenesis;assembly of protein complexes;protein binding;endocytosis;cell growth ;cell wall;actin cytoskeleton;budding;	192-202	83%
BZZ1	cytoplasm actin cytoskeleton	response to stress;osmotic and salt stress response;osmosensing;actin cytoskeleton;	MYO5	actin cytoskeleton	cell wall organization and biogenesis;conversion to kinetic energy ;cytoskeleton;exocytosis;endocytosis;osmotic and salt stress response;osmosensing;budding;	1008-1018	83%
BZZ1	cytoplasm actin cytoskeleton	response to stress;osmotic and salt stress response;osmosensing;actin cytoskeleton;	BCK1	cytoplasm	mitotic cell cycle and cell cycle control;modification by phosphorylation;MAPKKK cascade;stress response;perception of nutrients and nutritional adaptation;directional cell growth ;budding;	1394-1404	83%
BZZ1	cytoplasm actin cytoskeleton	response to stress;osmotic and salt stress response;osmosensing;actin cytoskeleton;	ARK1	cytoplasm actin cytoskeleton	cytokinesis;phosphate utilization;modification by phosphorylation;assembly of protein complexes;endocytosis;actin cytoskeleton;budding;	606-616	73%
BZZ1	cytoplasm actin cytoskeleton	response to stress;osmotic and salt stress response;osmosensing;actin cytoskeleton;	LAS17	cytoplasm actin cytoskeleton	cytokinesis;assembly of protein complexes;protein binding;endocytosis;osmotic and salt stress response;cell growth ;actin cytoskeleton;budding;	181-191	83%
BOI1	bud neck cell periphery cytoplasm	signal transduction;lipid binding;small GTPase mediated signal transduction;cell growth ;cytoskeleton;budding;	BCK1	cytoplasm	mitotic cell cycle and cell cycle control;modification by phosphorylation;MAPKKK cascade;stress response;perception of nutrients and nutritional adaptation;directional cell growth ;budding;	805-815	83%
BOI1	bud neck cell periphery cytoplasm	signal transduction;lipid binding;small GTPase mediated signal transduction;cell growth ;cytoskeleton;budding;	CDC54	cytoplasm nucleus	DNA metabolism;DNA topology;ori recognition and priming complex formation;mitotic cell cycle and cell cycle control;ATP binding;	680-690	83%
BOI1	bud neck cell periphery cytoplasm	signal transduction;lipid binding;small GTPase mediated signal transduction;cell growth ;cytoskeleton;budding;	RPO41	mitochondria mitochondria	organelle organization and biogenesis;transcription;DNA synthesis and replication;general transcription activities;DNA binding;mitochondrion;	828-838	73%
BOI1	bud neck cell periphery cytoplasm	signal transduction;lipid binding;small GTPase mediated signal transduction;cell growth ;cytoskeleton;budding;	LAS17	cytoplasm actin cytoskeleton	cytokinesis;assembly of protein complexes;protein binding;endocytosis;osmotic and salt stress response;cell growth ;actin cytoskeleton;budding;	310-320<br>321-331<br>348-358<br>381-391	83%
BOI1	bud neck cell periphery cytoplasm	signal transduction;lipid binding;small GTPase mediated signal transduction;cell growth ;cytoskeleton;budding;	STE20	bud cytoplasm	pseudohyphal growth;modification by phosphorylation;transmembrane signal transduction;pheromone response;cell growth ;cytoskeleton;budding;	473-483	83%
BOI1	bud neck cell periphery cytoplasm	signal transduction;lipid binding;small GTPase mediated signal transduction;cell growth ;cytoskeleton;budding;	BOI2	bud neck cell periphery cytoplasm	signal transduction;lipid binding;small GTPase mediated signal transduction;cell growth ;cytoskeleton;budding;	439-449	73%
RVS167	actin cytoskeleton	vesicle-mediated transport;response to stress;budding;protein binding;endocytosis;osmotic and salt stress response;cell growth ;cytoskeleton;budding;	ABP1	cytoplasm actin cytoskeleton	cytoskeleton organization and biogenesis;assembly of protein complexes;protein binding;cell growth ;actin cytoskeleton;budding;	515-525	73%

RVS167	actin cytoskeleton	vesicle-mediated transport;response to stress;budding;protein binding;endocytosis;osmotic and salt stress response;cell growth ;cytoskeleton;budding;	APP1	cytoplasm actin cytoskeleton nucleus	cytoskeleton organization and biogenesis;cell growth ;actin cytoskeleton;budding;	505-515	83%
RVS167	actin cytoskeleton	vesicle-mediated transport;response to stress;budding;protein binding;endocytosis;osmotic and salt stress response;cell growth ;cytoskeleton;budding;	LAS17	cytoplasm actin cytoskeleton	cytokinesis;assembly of protein complexes;protein binding;endocytosis;osmotic and salt stress response;cell growth ;actin cytoskeleton;budding;	341-351<br>384-394<br>384-394	83%
RVS167	actin cytoskeleton	vesicle-mediated transport;response to stress;budding;protein binding;endocytosis;osmotic and salt stress response;cell growth ;cytoskeleton;budding;	YDL146W	bud neck cell periphery cytoplasm nucleus	-	477-487	73%
MYO3	cytoplasm actin cytoskeleton	cell wall organization and biogenesis;conversion to kinetic energy ;cytoskeleton;exocytosis;endocytosis;osmotic and salt stress response;cell wall;budding;	SRV2	cell periphery actin cytoskeleton	cytoskeleton organization and biogenesis;metabolism of cyclic and unusual nucleotides;protein binding;small GTPase mediated signal transduction;cell growth;budding;	276-286	83%
MYO3	cytoplasm actin cytoskeleton	cell wall organization and biogenesis;conversion to kinetic energy ;cytoskeleton;exocytosis;endocytosis;osmotic and salt stress response;cell wall;budding;	FAR11	ambiguous	cell cycle;conjugation;pheromone response;	520-530	73%
MYO3	cytoplasm actin cytoskeleton	cell wall organization and biogenesis;conversion to kinetic energy ;cytoskeleton;exocytosis;endocytosis;osmotic and salt stress response;cell wall;budding;	BNI1	neck neck cytoplasm cytoskeleton	response to stress;budding;cytokinesis ;spindle pole body;protein binding;structural protein;RNA transport;small GTPase mediated signal transduction;osmotic and salt stress response;pheromone response;cell growth ;actin cytoskeleton;	1238-1248<br>1277-1287	83%
MYO3	cytoplasm actin cytoskeleton	cell wall organization and biogenesis;conversion to kinetic energy ;cytoskeleton;exocytosis;endocytosis;osmotic and salt stress response;cell wall;budding;	VRP1	cytoplasm actin cytoskeleton	vesicle-mediated transport;response to stress;budding;protein binding;endocytosis;osmotic and salt stress response;cell growth ;actin cytoskeleton;	4-14<br>234-244<br>395-405	83%
MYO3	cytoplasm actin cytoskeleton	cell wall organization and biogenesis;conversion to kinetic energy ;cytoskeleton;exocytosis;endocytosis;osmotic and salt stress response;cell wall;budding;	BNR1	neck cell periphery actin cytoskeleton	response to stress;budding;protein binding;osmotic and salt stress response;cell growth ;actin cytoskeleton;	765-775<br>819-829	83%
PIN3	cytoplasm nucleus	cytoskeleton organization and biogenesis;actin cytoskeleton;	APP1	cytoplasm actin cytoskeleton nucleus	cytoskeleton organization and biogenesis;cell growth ;actin cytoskeleton;budding;	506-516	83%
PIN3	cytoplasm nucleus	cytoskeleton organization and biogenesis;actin cytoskeleton;	LAS17	cytoplasm actin cytoskeleton	cytokinesis;assembly of protein complexes;protein binding;endocytosis;osmotic and salt stress response;cell growth ;actin cytoskeleton;budding;	324-334<br>342-352<br>353-363<br>385-395	83%
PIN3	cytoplasm nucleus	cytoskeleton organization and biogenesis;actin cytoskeleton;	GTS1	cytoplasm actin cytoskeleton nucleus	cell cycle;morphogenesis;mitotic cell cycle and cell cycle control;transcriptional activator;drug transport;stress response;rhythm ;cell growth ;cell aging;budding;development of asco;	248-258	73%
PIN3	cytoplasm nucleus	cytoskeleton organization and biogenesis;actin cytoskeleton;	YDL146W	bud neck cell periphery cytoplasm nucleus	-	478-488	73%
SLA1	cytoplasm actin cytoskeleton nucleus	cell wall organization and biogenesis;assembly of protein complexes;protein binding;endocytosis;cell growth ;cell wall;actin cytoskeleton;budding;	LAS17	cytoplasm actin cytoskeleton	cytokinesis;assembly of protein complexes;protein binding;endocytosis;osmotic and salt stress response;cell growth ;actin cytoskeleton;budding;	319-328<br>346-355<br>379-388	83%

SLA1	cytoplasm actin cytoskeleton nucleus	cell wall organization and biogenesis;assembly of protein complexes;protein binding;endocytosis;cell growth ;cell wall;actin cytoskeleton;budding;	STE20	bud cytoplasm	pseudohyphal growth;phosphate utilization;modification by phosphorylation;transmembrane signal transduction;pheromone response;cell growth ;cytoskeleton;budding;	471-480	83%

References

Dolinski, K., Balakrishnan, R., Christie, K. R., Costanzo, M. C., Dwight, S. S., Engel, S. R., Fisk, D. G., Hirschman, J. E., Hong, E. L., Nash, R., Oughtred, R., Theesfeld, C. L., Binkley, G., Lane, C., Schroeder, M., Sethuraman, A., Dong, S., Weng, S., Miyasato, S., Andrada, R., Botstein, D., and Cherry, J. M. "Saccharomyces Genome Database" UPftp://ftp.yeastgenome.org/yeast/UP

Mewes, H. W., D. Frishman, et al. (2000). "MIPS: a database for genomes and protein sequences." Nucleic Acids Res **28**(1): 37-40.
